# Effect of iron oxide and gold nanoparticles on bacterial growth leading towards biological application

**DOI:** 10.1186/1477-3155-9-34

**Published:** 2011-08-23

**Authors:** Saptarshi Chatterjee, Arghya Bandyopadhyay, Keka Sarkar

**Affiliations:** 1 Department of Microbiology, University of Kalyani, Nadia, West Bengal, India

**Keywords:** Bacterial Growth, magnetic nanoparticle, gold nanoparticle, Cytotoxicity

## Abstract

**Background:**

Nanoparticle-metal oxide and gold represents a new class of important materials that are increasingly being developed for use in research and health related activities. The biological system being extremely critical requires the fundamental understanding on the influence of inorganic nanoparticles on cellular growth and functions. Our study was aimed to find out the effect of iron oxide (Fe_3_O_4_), gold (Au) nanoparticles on cellular growth of *Escherichia coli *(*E. coli*) and also try to channelize the obtained result by functionalizing the Au nanoparticle for further biological applications.

**Result:**

Fe_3_O_4 _and Au nanoparticles were prepared and characterized using Transmission electron microscopy (TEM) and Dynamic Light Scattering (DLS). Preliminary growth analysis data suggest that the nanoparticles of iron oxide have an inhibitory effect on E. coli in a concentration dependant manner, whereas the gold nanoparticle directly showed no such activity. However the phase contrast microscopic study clearly demonstrated that the effect of both Fe_3_O_4 _and Au nanoparticle extended up to the level of cell division which was evident as the abrupt increase in bacterial cell length. The incorporation of gold nanoparticle by bacterial cell was also observed during microscopic analysis based on which glutathione functionalized gold nanoparticle was prepared and used as a vector for plasmid DNA transport within bacterial cell.

**Conclusion:**

Altogether the study suggests that there is metal nanoparticle-bacteria interaction at the cellular level that can be utilized for beneficial biological application but significantly it also posses potential to produce ecotoxicity, challenging the ecofriendly nature of nanoparticles.

## Background

The present era belongs to nanotechnology. With the tremendous growth in the field of science, nanobiotechnology has come up as a major interdisciplinary subject. The development and application of nanotechnology has the potential to improve greatly the quality of life. An improved understanding of nanoparticles and biological cell interaction can lead to the development of new sensing, diagnostic and treatment capabilities [1-4] such as improved targeted drug delivery, gene therapy, magnetic resonance imaging contrast agents and biological warfare agent detection [5,6]. For instance iron oxide nanoparticle has been widely used as carriers for targeted drug delivery to treat several types of cancer [7,8] in biomedical research because of its biocompatibility and magnetic properties [9,10]. Gold nanoparticle is the other mostly applied nanoparticle in the field of biomedical sciences expanding from immunoassay [11] to *in vivo *cancer targeting and imaging [12].

Though there are immense potentials of nanotechnology, the cytotoxicity of the nanoparticles remain a major concern. Different classes of bacteria exhibit different susceptibilities to nanoparticles [13] but the mechanism controlling the toxicity is not yet understood. Moreover different factors such as synthesis, shape, size, composition, addition of stabilizer etc can lead to different conclusions even for very closely related nanosuspensions [14]. Thus the present study is aimed to investigate the effect of two widely used nanoparticles (Fe_3_O_4_ & Au) on the growth of *E. coli*. The growth study was followed by microscopic study for detecting the morphological changes. Finally, attempts were made to utilize the results obtained for biological applications.

## Result and Discussion

### A) Characterization of nanoparticles

**Figure 1:**
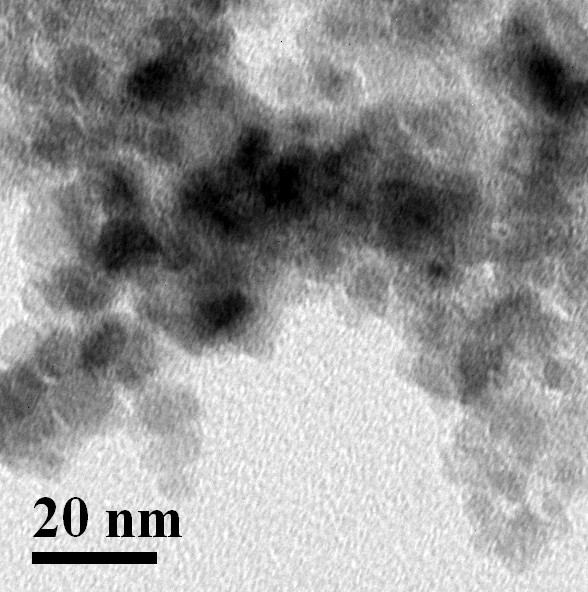
**Transmission Electron Microscope (TEM) image of Fe_3_O_4_ nanoparticle showing the size of the nanoparticle to be 8 nm (approx)**.

**Figure 2:**
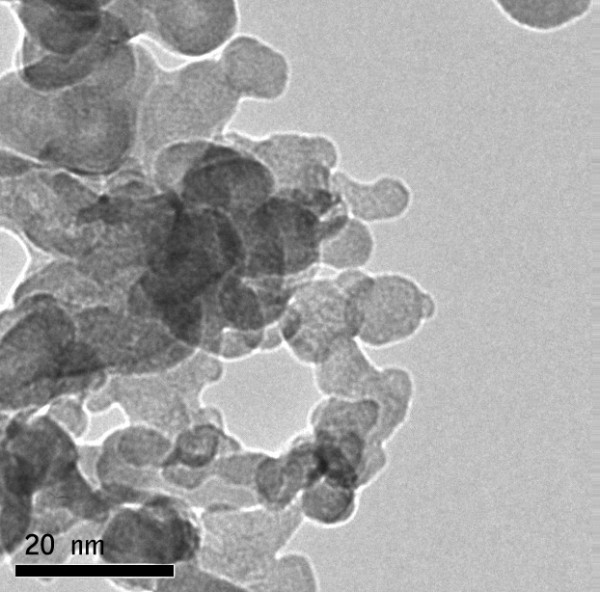
**Transmission Electron Microscope (TEM) image of Au nanoparticle showing the size of the nanoparticle to be 5 nm (approx)**.

**Figure 3:**
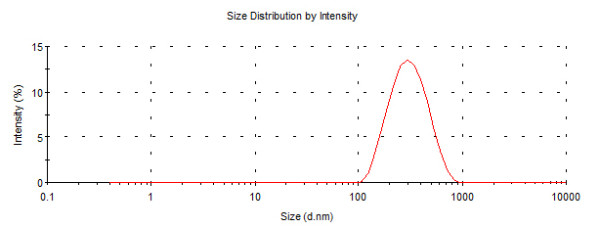
**Size distribution intensity graph of Fe_3_O_4_ nanoparticle as revealed by DLS**.

The nanoparticles (iron oxide & gold) synthesized in the laboratory were characterized using TEM image (FEI, Tecnai S-twin) and DLS (Malvern Zetasizer). The size of magnetic nanoparticle was found to be 8 nm by TEM image whereas Gold nanoparticle possessed size of 5 nm (Figure 1, 2). The DLS data of Fe_3_O_4_ and Au nanoparticles as shown in Figure 3, 4. indicated monodispersity.

**Figure 4:**
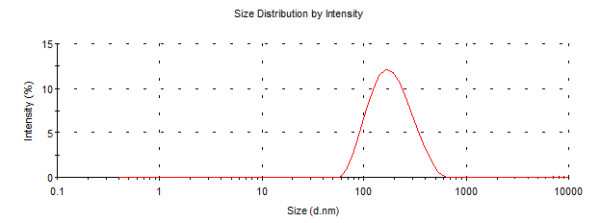
**Size distribution intensity graph of Au nanoparticle as revealed by DLS**.

### B) Effect of Iron nanoparticle on bacterial growth

**Figure 5:**
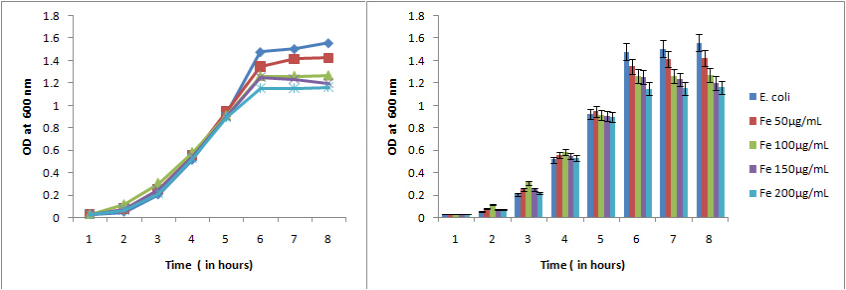
**Growth curve of *E. coli *under the influence of Fe_3_O_4_ nanoparticle compared to the normal growth curve of *E. coli *depicting the microbiocidal nature of the Fe_3_O_4_ nanoparticle in a concentration dependant manner**.

**Figure 6:**
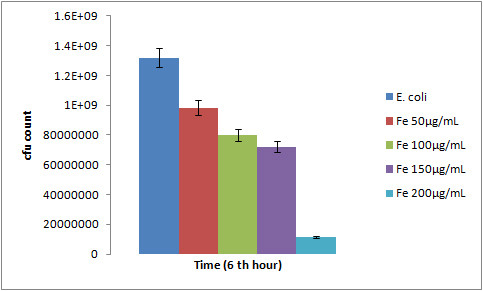
**Comparison of colony forming unit (cfu) count of *E. coli *(normal) and under the influence of Fe_3_O_4_ nanoparticle at 6th hour of bacterial growth**.

The comparative study on growth of bacteria under normal condition and under the influence of Magnetic nanoparticle (Fe_3_O_4_) revealed the effect of Fe nanoparticle on bacterial growth. The growth curve of *E. coli *under normal conditions clearly depicted the lag, log, stationary and death phase as shown in Figure 5 but under the influence of various concentrations of iron oxide nanoparticles (i.e 50 μg/mL, 100 μg/mL, 150 μg/mL & 200 μg/mL) the gradual shortening of log phase was evident indicating the microbiostatic effect of iron nanoparticle on *E. coli *in a concentration dependant manner. The untreated bacterial sample at 6th hour reached OD600 1.48 (cfu count 1.32 × 10^9^ per mL) compared to OD600 1.14 (cfu count 1.01 × 10^8^) in case of iron oxide (200 μg/mL) treated bacterial cells (Figure 6). The reactive oxygen species (ROS) along with superoxide radicals (O^2-^), hydroxide radical (OH^-^) and singlet oxygen (_1_O^2^) generated by the iron oxide nanopaticle is thought to be the reason behind the inhibition [15]. ROS production has been found in diverse range of metal oxide nanoparticles that may result in oxidative stress, inflammation and consequent damage to proteins, membranes and DNA which is one of the primary mechanisms of nanotoxicity.

### C) Effect of gold nanoparticle on bacterial growth

**Figure 7:**
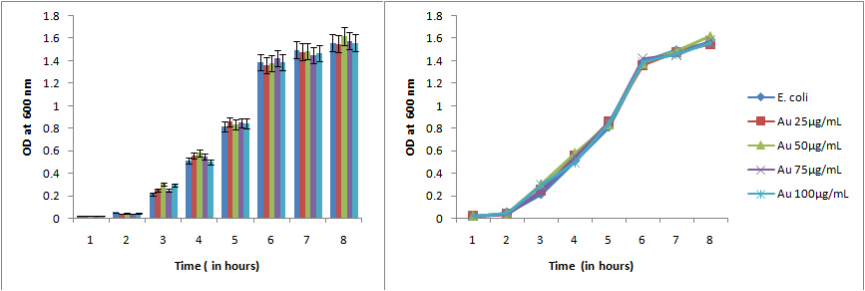
**Growth curve of *E. coli *under the influence of Au nanoparticle compared to the normal growth curve of *E. coli *indicating the nontoxic nature of Au nanoparticle**.

When *E. coli *was treated with various concentrations (25 μg/mL, 50 μg/mL, 75 μg/mL & 100 μg/mL) of gold nanoparticles no significant difference in the growth curve were obtained as shown in Figure 7. The growth experiment under the influence of gold nanoparticle thus reveals the nontoxic nature of the gold nanoparticle in the bacterial system (*E. coli*). Hence, it can be used for biological applications with least chances of cytotoxicity.

### D) Microscopic observation

**Figure 8:**
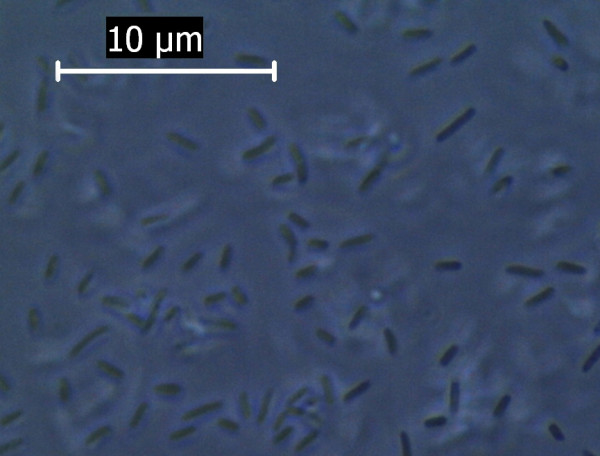
**Phase Contrast Microscopic image of *E. coli* grown under normal condition**.

**Figure 9:**
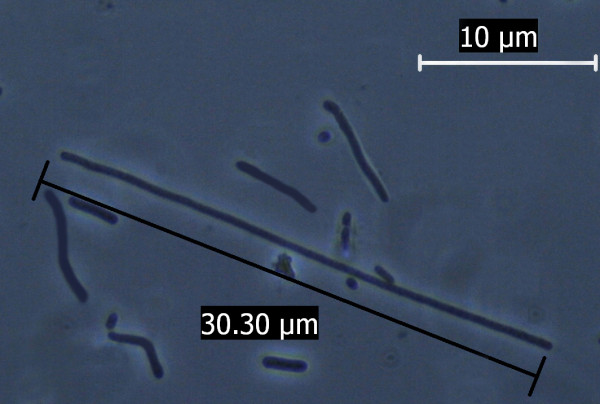
**Abrupt increase in *E. coli *cell length (up to 10 fold) grown under the influence of iron oxide nanoparticles, as observed under phase contrast microscope**.

**Figure 10:**
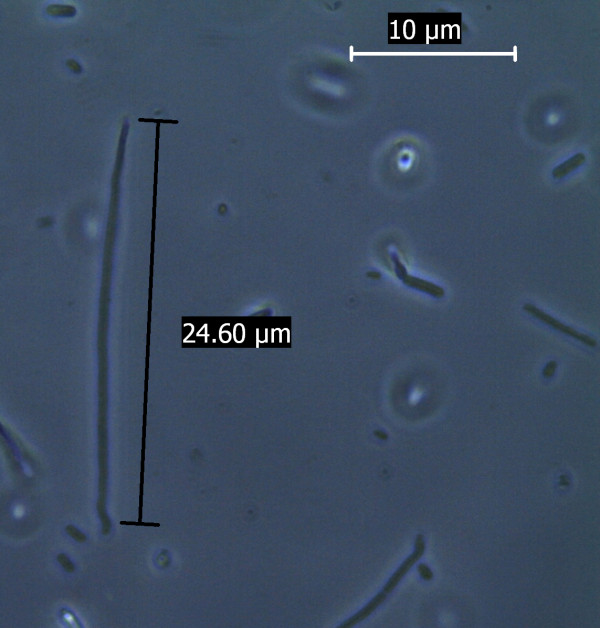
**Abrupt increase in *E. coli *cell length (up to 8 fold) grown under the influence of iron oxide nanoparticles, as observed under phase contrast microscope**.

**Figure 11:**
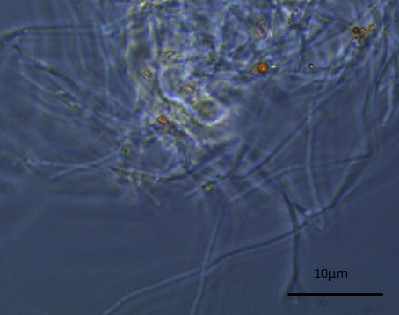
***E. coli *cells with abrupt cell length seen to be clogged in between the iron oxide nanoparticle when viewed under the phase contrast microscope**.

**Figure 12:**
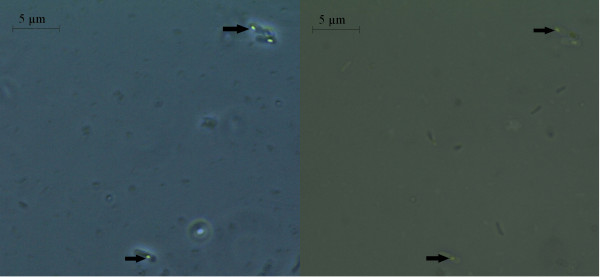
**Incorporation of Au nanoparticle was observed in the bacterial cell**.

The study was further extended at the microscopic level using phase contrast microscope. Both the nanoparticles were found to increase the size of the *E. coli *cell abruptly. The bacterial cell size under the influence of Fe_3_O_4_ nanoparticle when compared to that of normal *E. coli *cell (considering normal *E. coli *cell length to be approx. 3 μm as shown in Figure 8) showed up to a 10 fold increase in size Figure 9. The gold nanoparticle also gave identical result where the increase of cell length was up to 8 fold compared to that of normal *E. coli *cell as shown in Figure 10. The *E. coli *cells were also found to be clogged in between the iron oxide nanoparticles because of the magnetic property of the nanoparticle and the trapped cells also exhibited increased cell length (Figure 11). Iron oxide nanoparticles due to the high ionic strength frequently agglomerate in environmental and biological fluids, which shield the repulsion due to charges on the nanoparticles. Agglomeration has frequently been ignored in nanotoxicity studies, even though agglomeration would be expected to affect nanotoxicity since it changes the size, surface area, and sedimentation properties of the nanoparticles. Moreover nanoparticles can agglomerate to some extent in the environment or in the body before they reach their target; hence it is also desirable to study how toxicity is affected by agglomeration [16]. Thus our study indicates the effect of both the nanoparticles on the cellular level. Inactivation of certain gene expression required for 'cytokinesis' during cell division may be considered as a probable cause for such effect [17,18]. The result clearly shows the involvement of the nanoparticles on the bacterial physiology and is a probable demonstration of DNA nanoparticle interaction. The gold nanoparticle showed high tendency for incorporation within bacterial cells with the least possibility of cytotoxicity. This was evident during microscopic study, where grain like shining spots appeared within the bacterial cells (Figure 12).

### E) Biological Application of gold nanoparticle incorporation within bacterial Cells

As the incorporation of gold nanoparticle on *E. coli *cells were evident, studies were conducted to use this phenomenon for bio-applications. Since glutathione has an electrostatic interaction with both gold nanoparticle and DNA, the gold nanoparticle was surface modified using glutathione followed by interaction with plasmid DNA. The carboxyl group (COO^-^) of glycine residue electrostatically interacts with the positively charged gold nanoparticle to form glutathione functionalized gold nanoparticle. The other free end (γ-Glutamine residue) of glutathione now posses an amine group and a carboxyl group among which the amine group nonspecifically interacts with the negatively charged phosphate group of DNA forming a reversible electrostatic complex of gold-glutathione-DNA. This complex cleaves when incorporated within the bacterial cell due to ionic variation liberating the intact plasmid DNA from gold-glutathione complex. In our experiment the glutathione surface functionalized gold nanoparticles were used as a vector to insert ampicillin resistant gene (pUC 19) in *E. coli *that is susceptible to ampicillin. The result showed successful transformation of ampicillin resistant gene in *E. coli *as indicated by the growth of transformed bacteria in appropriate antibiotic containing media. The transformation efficiency was calculated as: Transformation efficiency = (Number of transformed colony/ Amount of DNA in μg ) and was found to be 8.53 × 10^5^ compared to that of 9.55 × 10^3^ using conventional CaCl_2_ mediated transformation. Thus we report glutathione functionalized gold nanoparticle mediated transformation as a bio-application for which further research is to be carried out to make this process generalized.

## Conclusion

Finally if we consider the recent past age to be of micro scale then the present or near future surely belongs to nano. Since most of the natural processes also take place in the nanometer scale therefore the association of nanotechnology and biology is expected to solve several biological problems. But the advances of the technology in the nanoscale level also remind the possible negative impact especially at the cellular level. From our research the interaction of two widely used nanoparticles with the bacterial cell was evident which opened a new dimension of biological application in the form of Au mediated transformation, though further research on the mechanism of interaction can reveal the further consequences which may open up a new domain of study called 'nanotoxicity'. However, as a cautionary note, the results presented are not meant to be generalized beyond the material and biological systems and conditions reported here.

Moreover our study proves the effect can be modified and channelized for human benefit.

Proper knowledge of these interactions can lead to a safe era of nanotechnology without threat of human health risk.

## Methods

### A) Preparation of Nanoparticles

#### i) Iron (Fe) Nanoparticle

Magnetic nanoparticles were prepared by chemical coprecipitation of Fe^2+^ and Fe^3+^ ions in an alkaline solution and followed by a treatment under hydrothermal conditions [19]. 2.7 g FeSO_4_, 7H_2_O and 5.7 g FeCl_3_ dissolved in 10 mL nanopure water (double distilled water filtered through 200 μm filter) separately. These two solutions was thoroughly mixed and added to double volume 10 M ammonium hydroxide with constant stirring at 25°C. Then the dark black slurry of Fe_3_O_4_ particles was heated at 80°C in a water bath for 30 min. The particles thus obtained exhibited a strong magnetic response. Impurity ions such as chlorides and sulphates were removed by washing the particles several times with nano pure water. Then the particles are dispersed in 20 mL nanopure water and sonicated for 10 min at 60 MHz. The yield of precipitated magnetic nanoparticles was determined by removing known aliquots of the suspension and drying to a constant mass in an oven at 60°C. The prepared magnetic nanoparticles were stable at room temperature (25-30°C) without getting agglomerated.

#### ii) Gold (Au) Nanoparticle

3 mM HAuCl_4_ solution was directly reduced by 10 mM NaBH_4_ solution under stirring condition. For further and complete reduction the reaction mixture was reduced again by 10 mg/ml solution of dextrose. Obtained mixture was subjected to over constant stirring. Then the mixture was washed several times with methanol using centrifugation at 65,000 rpm.

#### iii) Glutathione modified Gold (Au) Nanoparticle

Typically, 3.0 mM of glutathione was dissolved in 40 mL of distilled water, and 1.0 mM of HAuCl_4_ was dissolved in 80 mL of methanol. Mixing the two solutions generates a cloudy, white suspension. Addition of 10 mM of NaBH_4_ in 10 mL of water to this stirring suspension results in an immediate color change to dark brown indicating the generation of large cluster compounds. After additional stirring, the solution was evaporated at 43°C to near dryness and excess methanol was added to precipitate the clusters and wash reaction byproducts and any remaining starting material. The precipitate was then filtered and redissolved in 10 mL of distilled water, precipitated again with methanol, and filtered. These steps were repeated until a fine black powder was obtained [20].

### B) Growth Experiment

Test organism *Escherichia coli *(*E. coli*) were grown separately in 50 mL sterilized Luria Bertani (LB) broth medium and kept in shaker incubator at 37°C for 14 hour (overnight incubation). On the subsequent day test organism cultures were transferred at the rate of 1% in 100 mL LB broth kept in 250 mL conical flasks. Various concentrations of nanoparticles (For Fe_3_O_4_ 50 μg/mL to 200 μg/mL and for Au 25 μg/mL to 100 μg/mL) were carefully placed into each flask, leaving one as a control to track the normal growth of the microbial cells without nanoparticles. Experiments were performed using both a negative control (flask containing cells plus media) and a positive control (flask containing nanoparticles plus media). The flasks were shaken at 180 rpm and 37°C in a shaker incubator. Optical density measurements from each flask were taken every one hour to record the growth of the microbes in a spectrophotometer set at 600 nm. The growth rate of microbial cells interacting with the nanoparticles was determined from a plot of the log of the optical density versus time.

### C) Microscopic Study

The microscopic study on the morphology i.e the shape, size of the bacteria and interaction with the inorganic nanoparticles were conducted using Phase contrast microscope (Leica DM 750). 10 μL of culture was withdrawn every hour and microscopic study was conducted. The parameters were compared between normal culture and culture under the influence of inorganic nanoparticles (Fe_3_O_4_ & Au).

### D) Biological Application of Au Nanoparticle

As the property of internalization of Au nanoparticle within the *E. coli *cell was observed, the phenomenon was further investigated for its potential to be used for biological application. The insertion of Ampicillin resistant gene in the form of pUC 19 (Plasmid) was tried using the Au nanoparticles as vector/transport machinery. *E. coli *cells were grown on LB (Luria Bertani) broth till the O.D reaches 0.2. 10 μL of Au nanoparticles (50 μg/mL) were allowed to interact with 10 μL pUC 19 DNA (Bioserve, India) taken from a stock of 0.32 ng/μL for 2 hours at 37°C. Subsequently 1 mL of the *E. coli *culture (0.2 O.D) was centrifuged at 10,000 rcf for 1 min and 20 μL of pUC 19-Au nanoparticle mixture was added to the pellet. 980 μL of fresh LB medium was also added to it, mixed and incubated at 37°C for 5 hrs in shaking condition. Finally 100 μL of the culture were withdrawn and plated on Luria Bertani agar medium containing 50 μg/mL of ampicillin. The plates were incubated at 37°C overnight and numbers of colonies were counted. The cfu (Colony Forming Unit) count express the number of *E. coli *cells which posses the ampicillin resistant property acquired due to insertion of pUC 19 plasmid. The cfu count for the number of bacterial cells in the initial stage was also noted to compare the number of transformed cell to that of total bacterial cell. This efficiency of this method was also compared to that of conventional method [21] using CaCl_2 _mediated transfer of plasmid DNA in competent cells.

## Competing interests

The authors declare that they have no competing interests.

## Authors' contributions

**SC **carried out the growth experiments and biological application part whereas **AB **was engaged in the synthesis and characterization of nanoparticles. **KS **supervised in the design of the study along with critical interpretations while drafting the manuscript. All authors read and approved the final manuscript.

## Authors information

**S. Chatterjee: M.Sc. Microbiology**, Research Scholar, University of Kalyani.

**A. Bandyopadhyay: M.Sc. Microbiology**, Research Scholar, University of Kalyani.

**K. Sarkar: M.Sc., PhD**, Asst. Professor, Dept. of Microbiology, University of Kalyani.
